# Risk factors for fall among the elderly with diabetes mellitus type 2 in Jeddah, Saudi Arabia, 2022: a cross-sectional study

**DOI:** 10.1097/MS9.0000000000000269

**Published:** 2023-03-24

**Authors:** Rami S. Alasmari, Hattan A. Hassani, Nawwaf A. Almalky, Abdullah F. Bokhari, Abdullah Al Zahrani, Alwalied A. Hafez

**Affiliations:** a King Saud bin Abdulaziz University for Health Sciences, College of Medicine; b King Abdullah International Medical Research Center; c Department of Family Medicine, King Abdulaziz Medical City, Ministry of the National Guard – Health Affairs, Jeddah, Saudi Arabia

**Keywords:** diabetes, elderly, fall, risk factors

## Abstract

**Background:**

Diabetes mellitus type 2 is a major chronic condition that is considered common among elderly people, with multiple potential complications that could contribute to falls. However, this concept is not well understood; thus, the aim of this study is to estimate the prevalence of falls among diabetes patients.

**Methods:**

In this observational cross-sectional study, 309 diabetic patients aged 60 years or more who visited the primary healthcare centers of the Ministry of National Guard – Health Affairs in Jeddah were chosen via convenience sampling method. To collect the data, a structured Fall Risk Assessment questionnaire and Fall Efficacy Score scale were used.

**Results:**

The mean age of the participants was estimated to be 68.5 (SD: 7.4) years. Among the participants, 48.2% have fallen before, and 63.1% of them suffered falls in the past 12 months. The results showed that gait problems were independently associated with a higher likelihood of falls among elderly patients [odds ratio (OR)=1.98; 95% CI: 1.08–3.62; *P*=0.026]. Based on the linear regression analysis, we identified the following risk factors for lower falls efficacy: having gait problems (*β*=12.50; 95% CI: 7.38–17.6; *P*<0.001), balance difficulties (*β*=6.58; 95% CI: 1.35–11.8; *P*=0.014), and neurological/cognitive impairments (*β*=9.62; 95% CI: 3.89–15.4; *P*=0.001), as well as having poor sleep quality (*β*=8.11, 95% CI: 3.32–12.9; *P*<0.001).

**Conclusion:**

This paper suggests that diabetes mellitus is an independent fall risk factor among the elderly. Therefore, identifying such patients as being at higher risk and prompt referral to a specialist falls clinic is recommended.

Highlights

- Patients with diabetes who have a history of falls were notably older than those who are diabetic but never fell.
- Patients with diabetes who are receiving more than three medications are more at risk for falls than those who do not use any medications.
- The risk factors for falls for elderly diabetic patients were identified as having gait problems, balance difficulties, neurological and cognitive impairments, as well as having poor quality of sleep.

## Introduction

According to the WHO, a fall is defined as ‘an event which results in a person coming to rest inadvertently on the ground, floor or other lower level’^1^. In the United States of America, falls are a major cause of morbidity and mortality among the elderly^2^. Pursuing this further falls among the elderly can result in various adverse consequences, including higher incidence of hospitalization, health service use, loss of independence, decreased daily functioning, and greater fear of falling^3^,^4^. For someone to be described as elderly, they should have an age of 60 and above, according to the United Nations^5^. Unfortunately, Saudi Arabia does not have sufficient literature surrounding the topic of falls among the elderly. One study conducted in Riyadh calculated the annual prevalence rate of falling in the elderly group to be 49.9%, and linked risk factors were polypharmacy, walking aids, cerebrovascular accidents, retinopathy, and low back pain^6^. In addition, Alshammari *et al*.^7^ reported that age, female gender, impaired health, and environmental hazards were significantly associated with falls in 57.7% of the elderly participants of Riyadh. Moreover, a study in Unaizah city, Saudi Arabia, related to falling risk factors in the elderly, reported that the prevalence of obesity (with BMI>30) was 30.9%; furthermore, diabetes was estimated to be found to 18.2%, polypharmacy was found in 58%, and visual impairment, gait problems, memory loss, incontinence, and chronic pain were reported in 15.6% of elderly with falls^8^. Globally, ∼462 million individuals of all ages are diagnosed with diabetes mellitus type 2 (DMT2), corresponding to 6.28% of the world population. Moreover, in 2017 alone, there were more than 1 million deaths as a result of this condition ranking it as the ninth leading cause of mortality^9^. Saudi Arabia ranks the second highest in the Middle East and is seventh in the world for the rate of diabetes. Pursuing this further, it is estimated that around 7 million of the population are diabetic, and almost around 3 million have prediabetes^10^. It is well established that diabetes mellitus is associated with high mortality and multiple long-standing health complications such as microvascular complication including retinopathy and nephropathy, and macrovascular complications, such as peripheral vascular diseases resulting in injuries, nonhealing ulcers and gait disturbances ultimately resulting in lower limb amputation^11^. Nevertheless, the relationship between diabetes mellitus and falls in the elderly has not been studied in Saudi Arabia. Previous studies have demonstrated defined risk factors for falls (which included poor vision, balance difficulties, poor gait, motor weakness, sedatives, orthostatic hypotension, and others) among frail elderly^3^,^12^. Various potential complications from diabetes mellitus, such as diabetic retinopathy and peripheral neuropathy exhibiting as diabetic foot ulcer or inadequate glycemic control resulting in hypoglycemia, could be the cause of falls^5^,^13^,^14^. This is supported by many studies pointing out that diabetes mellitus might associate with falls among the elderly^5^,^13^–^15^. However, there is a knowledge gap regarding the risk factors for falls, particularly among the elderly with diabetes, since there is no such study that has been carried out in Saudi Arabia. Therefore, the aim of the present study is to identify the risk factors and to determine the relationship between falls and diabetes mellitus among the elderly.

## Material and methods

### Study participants, area, and period

A total of 309 diabetic patients aged 60 or more visited the primary healthcare centers of the Ministry of National Guard – Health Affairs in Jeddah, Saudi Arabia from May to June 2022. The inclusion criteria included 60 years of age or older Saudi patients with DMT@, and these participants were interviewed by five medical students for ∼10–30 min, and they answered questionnaires about the frequency of falls, the risk of falls, the activities of daily living, and cognition. In accordance with the Declaration of Helsinki, this study was registered in Research Registry with a unique identifying number which is researchregistry8607, and the study was approved by Institutional Review Board at King Abdullah International Medical Research Center #NRJ22J/097/04.

### Study design

This study employed a cross-sectional design through a population-based survey which was performed among elderly patients with diagnosed DMT2 in agreement with the STROCSS (Strengthening The Reporting Of Cohort Studies in Surgery) 2021 guidelines^16^.

### Instruments

Based on the proposed target population, who are type 2 diabetic patients at National Guard Hospital, we recruited an approximate number of 300 participants to be 95% confident with a 5% margin of error which is calculated using Raosoft.

The data were collected by a self-administered questionnaire that targets elderly Saudi patients with DMT2 at King Abdulaziz Medical City (KAMC), Jeddah, Saudi Arabia. The questionnaire was based on a validated questionnaire survey from previous studies^15^,^17^. Few modifications were made. First, the instruments used to collect data were a form addressing sociodemographic variables. Second, a structured Fall Risk questionnaire included a history of falls in the previous 1 year, medications, medical history, and environmental hazards. Lastly, the falls efficacy score (FES) scale in which its responses were recorded on a five-point Likert scale, ranging from strongly confident=1 to not confident at all=5. A raw FES was computed by summing up the values of the individual items (*n*=10). Therefore, the raw score ranged between 10 and 50. A percent FES was calculated to facilitate the interpretation of the results (range 20–100). Based on the responses of patients, higher FESs indicated lower fall self-efficacy.

### Statistical analysis

BMI was computed based on patients’ weights and heights, and the BMI values were then categorized into underweight (<18.5 kg/m^2^), healthy weight (18.5 to <25 kg/m^2^), overweight (25 to <30 kg/m^2^) and obese (≥30 kg/m^2^). The prevalence of falls was assessed using a one-sample proportions test, and the outcome was expressed as a proportion and 95% CI. Categorical data were presented as frequencies and percentages. Numerical data were expressed as mean±standard deviation (SD). To assess the association between the history of falls and sociodemographic and clinical variables, we used Fisher’s exact test or Pearson’s *χ* ^2^ test for categorical data and Wilcoxon rank sum test for numerical data. The significantly associated variables from the univariate analysis were subsequently used as independent variables in a multivariate logistic regression model to assess the risk factors for falls. The outcomes of the regression model were expressed as odds ratio (OR) and the respective 95% CI. To identify the risk factors for higher FES (lower self-efficacy), we carried out a multiple linear regression analysis using the backward stepwise method (FES was the dependent variable). This was performed by including all the potential independent variables at once (sociodemographic and clinical variables) and eliminating the least contributive variables until retaining the predictors that best fit the model. The results of the regression model were expressed as *β*-coefficient and 95% CIs. Statistical significance was considered at *P* less than 0.05. The analysis was performed using RStudio (version 4.1.1).

## Results

### Sociodemographic characteristics and the characteristics of fall

**Figure 1:**
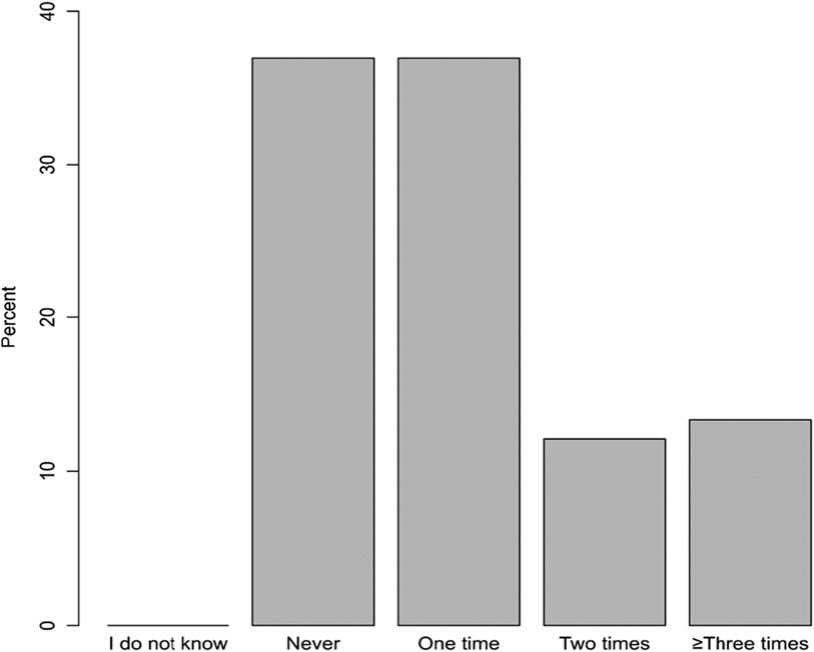
The proportions of responses regarding the frequency of falls in the last year among patients with a positive history of falls (*n*=149).

In the current study, data of 309 patients were analyzed. More than half of them were males (53.1%) and nonsmokers (68.6%). The majority of them were married (86.4%) and have at least one offspring (97.7%). Being overweight and obesity were prevalent among 35.3% and 47.9% of patients, respectively. A total of 149 patients (48.2%; 95% CI: 42.5–53.9%) indicated that they had never fallen before (Table 1). Focusing on patients who have fallen before, 36.9% of them have not fallen in the last year, 36.9% of patients have fallen once, 12.1% of them have fallen twice, and 13.4% have fallen three times, or more (Fig. 1). The mean±SD age of patients was 68.5±7.4 years, and patients who have a history of fall were significantly older than those who have never fallen (70.1±7.7 vs. 66.8±6.8 years; *P*<0.001). Other sociodemographic variables were not associated with the history of falls (Table 1).

**Table 1:** Sociodemographic characteristics of patients

Parameter	Category	Overall, *N*=309	No, *N*=160	Yes, *N*=149	*P*
Gender	Male	165 (53.2%)	85 (51.8%)	79 (48.2%)	0.985
	Female	145 (46.8%)	75 (51.7%)	70 (48.3%)	
Age (years)		68.5±7.4	66.8±6.8	70.1±7.7	<**0.0001**
Weight (kg)		80.2±17.2	80.5±18.6	79.9±15.7	0.921
Height (cm)		160.7±10.6	160.8±12.2	160.5±8.6	0.399
BMI	Underweight	2 (0.6%)	2 (100.0%)	0 (0.0%)	0.442
	Healthy	50 (16.1%)	29 (58.0%)	21 (42.0%)	
	Overweight	110 (35.5%)	53 (48.6%)	56 (51.4%)	
	Obese	148 (47.7%)	76 (51.4%)	72 (48.6%)	
Marital status	Divorced/separated	2 (0.6%)	1 (50.0%)	1 (50.0%)	0.452
	Married	268 (86.5%)	142 (53.2%)	125 (46.8%)	
	Widow/widower	40 (12.9%)	17 (42.5%)	23 (57.5%)	
Offspring (if married)*	Yes	302 (97.7%)	156 (51.8%)	145 (48.2%)	0.716
Smoking status	Never	213 (68.7%)	114 (53.8%)	98 (46.2%)	0.150
	Ex-smoker	63 (20.3%)	26 (41.3%)	37 (58.7%)	
	Current smoker	34 (11.0%)	20 (58.8%)	14 (41.2%)	

* The variable has one missing record.

Statistical significance at *P*< 0.05 values are in bold.

### The association between clinical factors of patients and fall

Significantly higher proportions of patients who have ever fallen had gait problems (64.8% vs. 28.7% among those who had no gait problems; *P*<0.0001), difficulties in the balance (69.8% vs. 35.4% among those who had no difficulties in the balance; *P*<0.0001), visual problems (52.6% vs. 31.1% among those who had no visual problems; *P*=0.003), musculoskeletal disorders (58.9% vs. 31.1% among those who had no musculoskeletal disorders; *P*<0.0001), and cardiovascular disease (51.0% vs. 35.1% among those who had no cardiovascular disease; *P*=0.034). A positive fall history was also associated with self-perceptions of an inappropriate surrounding environment (80.0% as inappropriate vs. 46.4% as appropriate; *P*=0.011) and a poor quality of sleep (66.7% vs. 34.3%% with good sleep quality; *P*<0.0001). Furthermore, a history of falls was associated with receiving less than 3 drugs (40.2%) and at least 3 drugs (54.0%) compared to patients who did not receive drugs (0.0%; *P*=0.002; Table 2). Upon incorporating the significantly associated variables into a regression model, results showed that only gait problems were independently associated with a higher likelihood of fall among the elderly patients (OR=1.98; 95% CI: 1.08–3.62; *P*=0.026; Table 3).

**Table 2:** The association between clinical factors of patients and falls

		Fall history			
Parameter	Category	Overall, *N*=309	No, *N*=160	Yes, *N*=149	*P*
Gait problems	No	143 (46.3%)	102 (71.3%)	41 (28.7%)	<**0.0001**
	Yes	165 (53.4%)	58 (35.2%)	107 (64.8%)	
	I do not know	1 (0.3%)	0 (0.0%)	1 (100.0%)	
Difficulties in balance	No	192 (62.1%)	124 (64.6%)	68 (35.4%)	<**0.0001**
	Yes	116 (37.5%)	35 (30.2%)	81 (69.8%)	
	I do not know	1 (0.3%)	1 (100.0%)	0 (0.0%)	
Visual problems	No	61 (19.7%)	42 (68.9%)	19 (31.1%)	**0.003**
	Yes	247 (79.9%)	117 (47.4%)	130 (52.6%)	
	I do not know	1 (0.3%)	1 (100.0%)	0 (0.0%)	
Neurological and/or cognitive impairments	No	255 (82.5%)	136 (53.3%)	119 (46.7%)	0.235
	Yes	54 (17.5%)	24 (44.4%)	30 (55.6%)	
Musculoskeletal disorders	No	119 (38.5%)	82 (68.9%)	37 (31.1%)	<**0.0001**
	Yes	190 (61.5%)	78 (41.1%)	112 (58.9%)	
Cardiovascular disease	No	57 (18.4%)	37 (64.9%)	20 (35.1%)	**0.034**
	Yes	251 (81.2%)	123 (49.0%)	128 (51.0%)	
	I do not know	1 (0.3%)	0 (0.0%)	1 (100.0%)	
Environment	Inappropriate	15 (4.9%)	3 (20.0%)	12 (80.0%)	**0.011**
	Appropriate	293 (94.8%)	157 (53.6%)	136 (46.4%)	
	I do not know	1 (0.3%)	0 (0.0%)	1 (100.0%)	
Number of medications	None	6 (1.9%)	6 (100.0%)	0 (0.0%)	**0.002**
	<3 drugs	102 (33.1%)	61 (59.8%)	41 (40.2%)	
	≥3 drugs	200 (64.9%)	92 (46.0%)	108 (54.0%)	
Quality of sleep	Good	175 (56.6%)	115 (65.7%)	60 (34.3%)	<**0.0001**
	Poor	132 (42.7%)	44 (33.3%)	88 (66.7%)	
	I do not know	2 (0.6%)	1 (50.0%)	1 (50.0%)	

Used with permission, Vahidreza Borhaninejad. Copyright; 2019.

Statistical significance at *P*< 0.05 values are in bold.

**Table 3:** Risk factors for falls among the patients

Parameter	Category	OR	95% CI	*P*
Age		1.01	0.98–1.05	0.425
Gait problems	No	–	–	
	Yes	1.98	1.08–3.62	0.026
	I do not know	NA	NA	0.994
Balance difficulties	No	–	–	
	Yes	1.82	0.98–3.37	0.057
	I do not know	NA	NA	0.994
Visual problems	No	–	–	
	Yes	1.39	0.71–2.79	0.343
	I do not know	NA	NA	0.995
Musculoskeletal disorders	No	–	–	
	Yes	1.67	0.93–2.99	0.083
Cardiovascular disease	No	–	–	
	Yes	1.36	0.68–2.76	0.383
	I do not know	NA	NA	0.994
Surrounding environment	Inappropriate	–	–	
	Appropriate	0.45	0.07–1.82	0.316
	I do not know	NA	NA	0.995
Number of medications	<3 drugs	–	–	
	None	NA	NA	0.986
	≥3 drugs	1.15	0.66–2.00	0.626
Sleeping description	Poor	–	–	
	Good	0.58	0.33–1.04	0.065
	I do not know	0.63	0.02–19.7	0.771

NA, not applicable because the variable has one zero frequency; OR, odds ratio.

Statistical significance at *P*< 0.05 values are in bold.

### Falls efficacy scale

The responses to the FES scale showed excellent internal consistency (Cronbach’s *α*=0.957); therefore, the results were reliable. The highest mean scores were related to getting on and off the toilet without falling (2.29±1.52) and preparing meals not requiring carrying heavy or hot objects (2.21±1.53), while the lowest scores were reported for personal grooming (1.63±1.16) and getting dressed and undressed (1.79±1.30; Table 4). Based on the linear regression analysis, we identified the following risk factors for lower falls efficacy: having gait problems (*β*=12.50; 95% CI: 7.38–17.6; *P*<0.0001), balance difficulties (*β*=6.58; 95% CI: 1.35–11.8; *P*=0.014) and neurological/cognitive impairments (*β*=9.62, 95% CI: 3.89–15.4; *P*=0.001), as well as having poor sleep quality (*β*=8.11; 95% CI: 3.32–12.9; *P*<0.0001; Table 5).

**Table 4:** Descriptive data of the fall efficacy score

Parameter	Mean±SD	Min–max
Take a bath or shower	1.97±1.43	1–5
Reach into cabinets or closets	2.09±1.50	1–5
Prepare meals not requiring carrying heavy or hot objects	2.21±1.53	1–5
Walk around the house	2.18±1.49	1–5
Get in and out of the bed	2.07±1.32	1–5
Answer the door or telephone	1.81±1.34	1–5
Get in and out of a chair	2.11±1.39	1–5
Get dressed and undressed	1.79±1.30	1–5
Personal grooming	1.63±1.16	1–5
Get on and off the toilet without falling	2.29±1.52	1–5
Total percent score	40.30±23.90	20–100

Used with permission, Mary E. Tinetti, M.D. Copyright; 2006.

**Table 5:** Risk factors for lower fall efficacy

Parameter	Category	*β*	95% CI	*P*
Age	Numeric	0.57	0.26–0.87	<**0.0001**
Gait problems	No	–	–	
	Yes	12.50	7.38–17.6	<**0.0001**
	I do not know	34.60	−1.77 to 71.0	0.062
Balance difficulties	No	–	–	
	Yes	6.58	1.35–11.8	**0.014**
	I do not know	19.80	−16.8 to 56.3	0.288
Neurological and/or cognitive impairments	No	–	–	
	Yes	9.62	3.89–15.4	**0.001**
Environment	Inappropriate	–	–	
	Appropriate	−7.56	−17.6 to 2.47	0.139
	I do not know	33.10	−4.31 to 70.5	0.083
Number of medications	Not using any drug	–	–	
	Less than three drugs	2.20	−13.3 to 17.7	0.780
	Three drugs or more	7.54	−7.80 to 22.9	0.334
Quality of sleep	Good	–	–	
	Poor	8.11	3.32–12.9	<**0.0001**
	I do not know	−20.00	−45.9 to 5.85	0.129

Statistical significance at *P*< 0.05 values are in bold.

## Discussion

The aim of the present study was designed to determine the risk factors of falls in elderly diagnosed with diabetes mellitus and its association. The significant finding of this study was found to be that a total of 149 patients representing ∼48.2% have experienced falling before. Among this population, there is nearly 63.1% who have fallen in the past 1 year. As previously indicated, fall incidents among the elderly are a major health problem. About 36 million falls are reported among the elderly each year, resulting in more than 32 000 deaths^18^. Consistent with findings by Montero-Odasso *et al*.^19^, we found that the elderly with gait problems are at higher risk of developing mobility decline and falls. A continuous gait observation that provides fall risk assessment would permit timely interventions aiming at preventing falls^20^. Step training, therefore, is a key component of fall prevention interventions^21^. Difficulties in balance was one of the findings suggesting risk factor for falls. Multiple studies done in the United States, Brazil, and Canada showed an agreement with this study results and pointed out that decreased fear of falling, balance and strength promote the elderly quality of life and independence^22^–^24^. In the current paper, the purpose of using FES scale is to determine the extent to which fear of falling exhibits an independent effect on the functional decline among the elderly. The responses to the FES scale showed excellent internal consistency (Cronbach’s *α*=0.957); therefore, the results were reliable. Based on the linear regression analysis, having gait problems, balance difficulties, neurological/cognitive impairments, as well as having poor sleep quality were determined to be significant risk factors for lowering fall efficacy, thus increasing fear of falling. If the fear of falling confirms to be an independent factor in functional decline, and if the person at risk of developing a fear of falling can be recognized, then the fear of falling efficacy should be a specific target of clinical intervention^17^. In fact, Bandura *et al*.^25^ and Soh *et al*.^26^ state that self-efficacy has been shown to be amenable to behavioral modification. Moreover, physical and occupational therapy could be targeted at promoting confidence in mobility and performance of daily activities^27^,^28^. Our study reveals that elderly diabetic patients are more prominent to experience falling (Fig. 1). Moreover, the prevalence of falls increased as the patients complain of gait problems (Table 3). Unfortunately, there are a few studies that have been carried out to discuss the relationship between elderly diabetic patients and falls. However, a prospective cohort study held in the United States showed agreement with our result by indicating that the fall incidence rate for elderly residents of a long-term care facility with and without diabetes mellitus was 78% and 30%, respectively, considering gait and balance as an independent predictor of falls^29^. Another study done in the United Kingdom displays that 39% of elderly diabetic patients report falling each year^30^. Roman de Mettelinge *et al*.^31^ and Schwartz *et al*.^32^ demonstrate that diabetes mellitus might be an independent risk factor for falls in elderly diabetic patients. The prevalence of falls in elderly diabetic patients increased as the frequency of hypoglycemia increased, according to a study held in Japan^33^. Finally, according to the literature and the present study, the authors believe that elderly diabetic patients are more prone to experience falling, and the probability of that would increase if the patients complained of an associated gait problem, balance difficulties, and hypoglycemic attacks. There are few limitations in our study that need to be addressed. First, the current study was an observational cross-sectional study. A prospective study will be indispensable to illustrate the relationship between balance difficulties, gait problems, and hypoglycemic attacks with risk of falls. Furthermore, our sample size was limited and collected conveniently. Hence, a large-scale multicenter study with larger sample size will be needed. Additionally, medications were restricted to only numerals. The medications need to be specified and classified as they might be causative for hypoglycemia, insulin, for instance.

## Conclusion

To sum up, this paper demonstrated that gait problems and balance difficulties, to some extent, are independent risk factors for falls in elderly diabetic patients. Not only having gait problems and balance difficulties but also neurological/cognitive impairments as well as poor sleep quality were confirmed to lower fall efficacy, thus increasing fear of falling. In conclusion, diabetes mellitus, defined by the use of hypoglycemic agents, is an independent risk factor for falls among the elderly. Pursuing this further, it should be considered a risk factor for falls in this population. In fact, recognizing such patients as being at higher risk could assist some preventive measures targeted at reducing the risk of falls.

## Ethical approval

Ethical approval (reference number: NRJ22J/097/04) was granted by the Institutional Review Board of King Abdullah International Medical Research Center, Jeddah, Saudi Arabia. This study was conducted in accordance with the principles of the Declaration of Helsinki.

## Patient consent

Informed written consent was obtained at the start of the questionnaire.

## Sources of funding

There were no sources of funding received for the current study.

## Author contribution

A.A.Z.: contributed to the proposal and manuscript writing and editing; R.S.A.: was involved in proposal and manuscript writing in addition to data collection and analysis; N.A.A., H.A.H., and A.F.B.: were involved in proposal and manuscript writing in addition to data collection; A.A.H.: contributed in revising the questionnaire and the manuscript. All authors granted approval of the final version of the manuscript.

## Conflicts of interest disclosure

The authors declare no conflicts of interest.

## Research registration unique identifying number (UIN)

1. Name of the registry: Research Registry http://www.researchregistry.com
2. Unique identifying number or registration ID: researchregistry8607.
3. Hyperlink to your specific registration (must be publicly accessible and will be checked): https://www.researchregistry.com/browse-the-registry#home/registrationdetails/63aef7688eaf0a0011ee3d5d/

## Provenance and peer review

Not commissioned, externally peer-reviewed.

## Guarantor

Rami S. Alasmari, King Saud bin Abdulaziz University for Health Sciences, College of Medicine, Jeddah, Saudi Arabia. Tel: +966 593 908 385. E-mail: alasmari097@ksau-hs.edu.sa, rami-1419@hotmail.com. Address: Jeddah, Alssamer District, Mendad Alkoukhi Street, Postcode 23462.
